# Foaming of 3D-Printed PLA/CaCO_3_ Composites by Supercritical CO_2_ Process for Sustainable Food Contact Materials

**DOI:** 10.3390/polym16060798

**Published:** 2024-03-13

**Authors:** Simón Faba, Ángel Agüero, Marina P. Arrieta, Sara Martínez, Julio Romero, Alejandra Torres, María José Galotto

**Affiliations:** 1 Packaging Innovation Center (LABEN-CHILE), Department of Food Science and Technology, Faculty of Technology, Center for the Development of Nanoscience and Nanotechnology (CEDENNA), University of Santiago de Chile (USACH), Santiago 9170201, Chile; sara.martinez@usach.cl (S.M.); alejandra.torresm@usach.cl (A.T.); maria.galotto@usach.cl (M.J.G.); 2 Departamento de Ingeniería Química Industrial y del Medio Ambiente, Escuela Técnica Superior de Ingenieros Industriales, Universidad Politécnica de Madrid (ETSII-UPM), Calle José Gutiérrez Abascal 2, 28006 Madrid, Spain; anagrod@epsa.upv.es; 3 Instituto Universitario de Tecnología de Materiales (IUTM), Universidad Politécnica de Valencia (UPV), Plaza Ferrándiz y Carbonell 1, 03801 Alcoy, Spain; 4 Grupo de Investigación: Polímeros, Caracterización y Aplicaciones (POLCA), 28006 Madrid, Spain; 5 Laboratory of Membrane Separation Processes (LabProSeM), Department of Chemical Engineering and Bioprocesses, Engineering Faculty, University of Santiago de Chile (USACH), Santiago 9170201, Chile; julio.romero@usach.cl

**Keywords:** poly(lactic acid), 3D printing, foams, supercritical CO_2_

## Abstract

In the last decade, among the emerging technologies in the area of bioplastics, additive manufacturing (AM), commonly referred to as 3D printing, stands out. This technology has gained great interest in the development of new products, mainly due to its capability to easily produce customized and low-cost plastic products. This work aims to evaluate the effect of supercritical foaming of 3D-printed parts based on a commercial PLA matrix loaded with calcium carbonate, for single-use sustainable food contact materials. 3D-printed PLA/CaCO_3_ parts were obtained by 3D printing with a 20% and 80% infill, and two infill patterns, rectilinear and triangular, were set for each of the infill percentages selected. Supercritical fluid foaming of PLA/CaCO_3_ composite printed parts was performed using a pressure of 25 MPa, a temperature of 130 °C for 23 min, with a fast depressurization rate (1 s). Closed-cell foams were achieved and the presence of CaCO_3_ did not influence the surface of the foams or the cell walls, and no agglomerations were observed. Foam samples with 80% infill showed subtle temperature fluctuations, and thermogravimetric analysis showed that samples were thermally stable up to ~300 °C, while the maximum degradation temperature was around 365 °C. Finally, tensile test analysis showed that for lower infill contents, the foams showed lower mechanical performance, while the 80% infill and triangular pattern produced foams with good mechanical performance. These results emphasize the interest in using the supercritical CO_2_ process to easily produce foams from 3D-printed parts. These materials represent a sustainable alternative for replacing non-biodegradable materials such as Expanded Polystyrene, and they are a promising option for use in many industrial applications, such as contact materials.

## 1. Introduction

Additive manufacturing (AM), commonly referred to as 3D printing, has garnered significant attention in the realm of new product development. This is primarily due to its cost-effectiveness and accessibility, making it a technology that can effortlessly produce affordable and customized products featuring geometrical complex structures [1,2]. Additionally, it includes online co-creation environments in which several documents of already designed products are shared by the “makers” movement [3,4].

Fused Deposition Modeling (FDM) is the most widely extended AM technique among others and is mainly used to process thermoplastic polymers. It also is one such method that involves techniques applied to build physical parts or complex structures following a layer-by-layer approach [5,6]. FDM stands out as the predominant 3D-printing method owing to its rapid and adaptable printing process, as well as its versatility in design and the wide variety of polymeric-based filament options (i.e., thermoplastic composites and nanocomposites) that are fused and further deposited by sequential 2D paths conforming the layers to obtain three-dimensional products [4,7,8]. However, as 3D-printed parts with a great number of layers on the Z axis are restricted in FDM, in some cases, 3D-printed parts can present low mechanical and dimensional stability of the layer-by-layer growth throughout the 3D-printing process, making necessary a post-processing treatment (i.e., chemical crosslinking, sintering if contain metals, among others) [9,10].

In this regard, polylactic acid (PLA) is the most widely used biobased and biodegradable polymer for 3D printing due to its versatile printing properties through the use of suitable additives [11,12,13]. PLA is a commercially available thermoplastic biopolyester made from renewable resources and is biodegradable. PLA is a commercially promising sustainable polymer as an alternative for replacing conventional non-renewable materials, such as LDPE (low-density polyethylene), PS (polystyrene), and PET (polyethylene terephthalate) in many fields of applications [14,15,16]. Moreover, it is compostable and biocompatible, can be obtained from various natural feedstock, and can be further degraded under composting environments by a hydrolysis process, which is then followed by the attack of microorganisms [17,18].

In this context, PLA can be processed through conventional thermoplastic technologies readily accessible in the plastics processing industry. These include melt-extrusion, film formation, thermoforming, injection molding, and foaming processes [19]. Conversely, PLA exhibits some limitations regarding its thermo-mechanical and barrier properties. To improve these limitations and extend the PLA applications in the market, its crystallinity is usually increased through the development of polymeric blends [18] and/or the use of filler or nanofillers to obtain composites or nanocomposites, respectively [20,21]. The most effective approach for enhancing the performance of 3D-printed products derived from PLA through the FDM approach involves formulating PLA composite materials with sustainable additives [22]. In this sense, PLA loaded with calcium carbonate (CaCO_3_), PLA composite, has been extensively studied and it has been proven that the addition of CaCO_3_ significantly enhances the mechanical performance of PLA [23,24,25]. As the CaCO_3_ content rises in the formulation, there is an increase in the Young’s modulus of the composites, coupled with a reduction in both tensile strength and elongation at break [26]. Additionally, PLA/CaCO_3_ composites have been studied for their potential use in the clinical setting in medicine [27]. Even its potential use as a material for lightweight construction solutions has been demonstrated, as the CaCO_3_ addition allows the production of materials with four times the insulation of gypsum [28].

Moreover, the development of PLA foams represents a promising and innovative alternative for the development of active materials for food contact applications [29,30,31]. The production of PLA foams can be conducted using chemical agents, batch processing with supercritical carbon dioxide (scCO_2_), extrusion foaming, or foam injection molding [32,33]. On this basis, PLA foams have recently emerged as an alternative in petrochemical-based food contact applications and other single-use and/or fast-moving consumer goods, being able to replace conventional polyolefin-based polymeric foams such as those based on polystyrene (PS), as PLA-based products are of renewable origin and offer a more sustainable end-of-life option because they are compostable [34,35,36].

Although 3D-printed products have attracted significant attention throughout the last few years and many research works have been published in this field, to the best of our knowledge, there are no works in which the properties of PLA-based 3D-printed parts foamed with scCO_2_ have been studied. Thus, in this work, as an alternative in the field of single-use sustainable food contact materials as a replacement for expanded PS foams, the effect of supercritical foaming of 3D-printed parts based on a commercial PLA matrix loaded with calcium carbonate was evaluated. Therefore, the properties of 3D-printed PLA foamed materials were evaluated and compared with 3D-printed pieces using two widespread printing patterns, linear and triangular, as well as two percentages of infill, 20% and 80%. The analysis of both 3D-printed parts and foams involved the assessment of mechanical (tensile test) and thermal properties through techniques such as thermogravimetric analysis (TGA) and differential scanning calorimetry (DSC). Surface chemical alterations in specific functional groups were examined using Fourier-transform infrared spectroscopy (FTIR) while scanning electron microscopy (SEM) was employed to investigate the microstructure of the generated foams. The findings underscore the promising application of supercritical carbon dioxide (scCO_2_) in facilely producing foams from 3D-printed components, with potential relevance in diverse sectors, including those involving food contact applications.

## 2. Materials and Methods

### 2.1. Materials

The 3D-printing filament under the tradename Smarfil^®^ E.P (Limestone), was provided by Smart Materials 3D Printing S.L. (Alcalá la Real, Spain). It is a material composed of 70 wt.% of PLA and 30 wt.% of Calcium Carbonate—CaCO_3_ [37]. PLA filament is made of high-quality PLA, without recycled material, and commercialized in coins of 1.75 mm diameter filament. It is an easy-to-use 3D filament in a wide range of 3D printers since it prints at low temperatures (±200 °C) and does not present a warping effect.

### 2.2. 3D Printing

**Figure 1:**
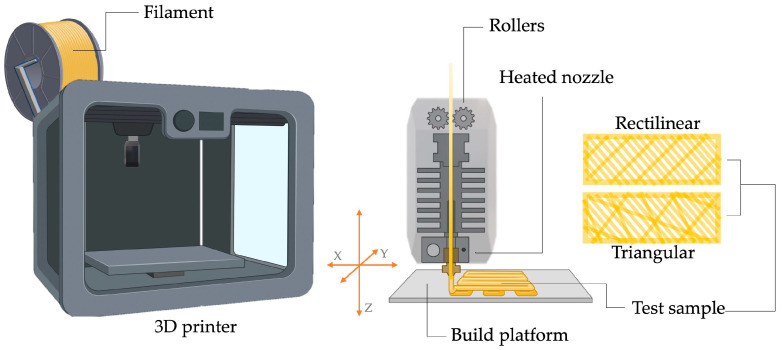
Schematic depiction of the experimental arrangement used for the 3D-printing process.

PLA samples were fabricated by FDM using a commercial 3D printer, Figure 1 (BQ Witbox 3D printer, BQ company, Madrid, Spain) equipped with a 1.75 mm diameter filament coin of the above-mentioned Smarfil^®^ PLA. The parameters employed for the 3D printing of the samples are outlined in Table 1. The filament was introduced into the heated nozzle via a roller feed mechanism. Following this, the material underwent melting and extrusion through the nozzle, with the molten material being deposited into a reservoir at room temperature.

Generally, 3D printing parts with less than 20% infill results in flimsy parts, whereas more than 50–60% infill is recommended when the wholeness of the piece is very relevant, due to the longer printing time needed. For printing specific parts that may be fixed by screws, drilled and so on, the infill percentage can be increased up to 80% [1,38]. In this work, to verify the feasibility of obtaining foamed materials from printed parts with generic printing parameters, two different infill percentages were set. Specifically, two extreme values were selected (20% and 80%). Due to the diffusion phenomenon that occurs in CO_2_ supercritical foaming, the infill pattern in which the filament was arranged during the print can directly influence the characteristics of the resulting foam. Hence, two different and very commonly used infill patterns were set for each of the infill percentages selected. All these parameter combinations that define each type of piece that has been used for foaming are listed in Table 1 while the printing process of the different parts is schematized in Figure 1.

To print all the parts with the different combinations, a single rectangle 3D model was used (40 × 10 × 0.6 mm^3^), developed in CAD software (Autodesk 3D Max software 2015, Autodesk Inc., San Rafael, CA, USA, EEUU). Once the 3D model was exported as an STL type file, the model slicing and setting of the printing parameters were carried out by using open-access software (UltiMaker Cura 5.3.1, Ultimaker B.V., Geldermalsen, The Netherlands). The main operational printing parameters set are gathered in Table 2.

### 2.3. Supercritical Foaming of 3D-Printed PLA Samples

**Figure 2:**
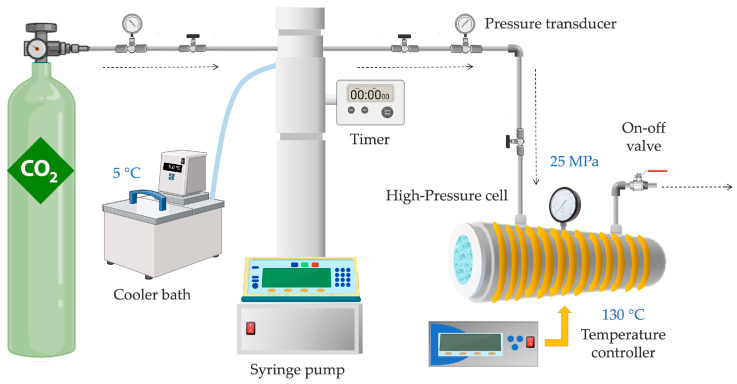
Diagram of the experimental arrangement for the supercritical foaming process.

The supercritical fluid foaming of PLA/CaCO_3_ composite printed parts was obtained employing a system outlined schematically in Figure 2. For that, a 100 mL high-pressure cell was utilized, and scCO_2_ was introduced using a Teledyne^®^ ISCO 260D high-pressure pump, as well as an ISCO D-Series syringe pump (Lincoln, NB, USA). The operation involved maintaining a constant pressure rate throughout the foaming runs. The temperature of the syringe pump was controlled by a bath cooler connected to a blanket around the pump’s compression cylinder, maintained at a consistent and controlled temperature of 5 °C, this allowed more liquid CO_2_ to enter the high-pressure cell. The experiments were conducted under consistent pressure (25 MPa) and temperature (130 °C) conditions for a duration of 23 min, on the basis of previous works and the results reported by Jeong et al. [39]. The system temperature was regulated using a thermostatic electric resistance encircling the cell. Following the saturation of the PLA parts with scCO_2_, the high-pressure cell underwent depressurization to atmospheric pressure by rapidly releasing CO_2_ within 1 s. The system was stabilized by convective air cooling at room temperature (approximately 25 °C ).

### 2.4. 3D-Printed Parts and Foams Characterization

#### 2.4.1. Intrinsic Viscosity and Viscosity Molecular Weight

The intrinsic viscosity [*η*] of the PLA bionanocomposite 3D-printed parts and foams was determined by assessing its capillary viscosity using a Ubbelohde viscometer (type C) according to the guidelines of ISO 1628 [40]. The specimens were diluted in chloroform (Sigma-Aldrich 99% purity) and filtered using glass filtering crucibles with a pore diameter of 16 to 40 µm [41]. The viscometer was immersed in a water bath to maintain a constant temperature of 25 °C during the assessment. At least five concentrations of each sample were employed and the linear regression of each intrinsic viscosity plotted against the concentration value was obtained. Subsequently, the viscosity molecular weight (*M_v_*) was estimated using the Mark–Houwink equation (Equation (1)). As the standard indicates, the [*η*] value employed for each specimen in Equation (1) is the theoretical value obtained by the cut of the linear regression and the ordinate axis.

(1) $$\left[\eta \right]=K\times M_{v}^{a}$$

where *K* and a are 1.53 × 10^−^^2^ and 0.759 respectively, for PLA [41].

#### 2.4.2. Microstructural Characterization 

The cellular microstructure of foamed 3D-printed samples was examined using scanning electron microscopy (SEM). Prior to analysis, the 3D-foamed parts were cryofractured by freezing them in liquid nitrogen and then coated with a gold alloy layer using a Q150 metallizer (Quorum Technologies, Sussex, UK). Samples were observed by JEOEL 6400 scanning electron microscopy (SEM) (Tokyo, Japan), with an accelerating voltage of 25 kV. To ascertain the distribution of cell sizes, a minimum of 100 cells located in the central zone of the cross-section of each fractured foam were measured through digital image analysis employing the ImageJ software (version 1.48v) [36].

#### 2.4.3. Fourier Transform Infrared (FTIR-ATR) Spectroscopy

ATR-FTIR spectra of the obtained foams and 3D-printed PLA parts were recorded by means of a Bruker Alpha spectrometer (Wismar, Germany) well-appointed with an attenuated total reflection diamond crystal accessory (Bruker, Platinum). ATR-FTIR curves were aimed to characterize the presence/absence of particular chemical groups within the obtained 3D parts and foams. The spectra were acquired with a resolution of 4 cm^–1^ in a wavenumber range from 4000 to 400 cm^–1^, utilizing 100 scans. Spectrum analysis was conducted by means of OPUS software version 7 (Bruker, Ettlingen, Karlsruhe, Germany).

#### 2.4.4. Thermal Properties

Differential Scanning Calorimetry (DSC) analysis was conducted using a Mettler Toledo DSC 822e International colorimeter (Schwerzenbach, Switzerland). Thermal analysis experiments were carried out to investigate the impact of the foaming process on the modification of the polymeric crystalline structure. Aluminum capsules were filled with 5.0 to 8.0 mg of 3D parts per run. Meanwhile, the capsules for foamed samples were prepared with 1.0 to 2.0 mg of samples mainly because of the low density of the foams. The samples underwent heating from 25 to 200 °C at a rate of 10 °C min^−1^ under a nitrogen atmosphere to prevent thermo-oxidative degradation.

Thermogravimetric analysis (TGA) assays were conducted in a Mettler Toledo Gas Controller GC20 Stare System TGA/DCS (Schwarzenbach, Switzerland). Samples were subjected to heating from 30 to 600 °C at a rate of 10 °C min^−1^ under a nitrogen atmosphere with a flow rate of 50 mL min^−1^ to prevent thermo-oxidative degradation of the PLA samples. The onset decomposition temperature (T_onset_) at 5% mass loss, while the maximum degradation temperature (T_max_) values were taken from the first derivative curve. TGA analysis facilitated the identification of degradation type and the assessment of thermal stability across the PLA foam formulations.

#### 2.4.5. Tensile Properties

The mechanical features were assessed through tensile test measurements conducted on a Shimadzu AGS-X universal tensile testing machine (Shimadzu Corporation, Kyoto, Japan) equipped with a 100 N load cell. At least five rectangular samples sized 5 × 50 mm^2^ of each foam were tested at a crosshead speed of 10 mm min^–1^ and an initial length of 30 mm. The stress–strain curves generated were used to calculate the average values of the Young’s modulus (E), tensile strength (TS), as well as percentage elongation at break (ε_b_%).

### 2.5. Statistical Analysis

Data analyses were performed using Statgraphics Plus 5.1 (StatPoint Inc., Herndon, VA, USA). The software was utilized for conducting variance analysis through an ANOVA test. The experimental design was random in nature. Statistical significance was established at a *p*-value of <0.05.

Data analyses were performed using Statgraphics Plus 5.1 (StatPoint Inc., Herndon, VA, USA). This software was utilized for conducting variance analysis through an ANOVA test. The experimental design adopted was random-type. Statistical significance was established at a *p*-value < 0.05.

## 3. Results

### 3.1. Processing of PLA Parts and Foams

**Figure 3:**
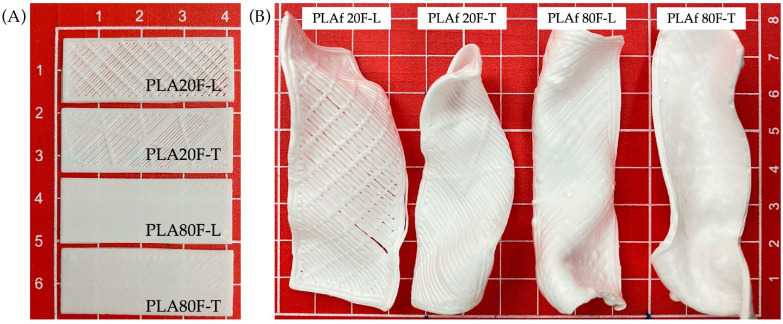
3D-printed (**A**) PLA/CaCO_3_ parts and (**B**) foams.

PLA parts with different infill percentages and print patterns were prepared by the FDM process followed directly by the scCO_2_ foaming process, as it was defined in Section 2.2. In Figure 3A, the printed samples are shown, and as one can see, for the sample with the 20% infill the inner pattern of each of them (linear and triangular) can be observed. Otherwise, those with 80% infill did not show the inner pattern. The 3D-printed PLA parts underwent foaming assisted by supercritical carbon dioxide (scCO_2_), as detailed in Section 2.3. The temperature was evaluated between 100 °C and 135 °C, while the system pressure varied within the range of 10 to 25 MPa. Initial tests yielded a scCO_2_ saturation rate at 22 min for samples with 20% fill and 24 min for those with 80% fill. The processing temperature was 130 °C and pressure was maintained at 25 MPa. The depressurization rate was maintained at a consistent 1 s. Figure 3B displays photographs of the obtained PLA/CaCO_3_ foams in which it can be seen that foamed samples present asymmetric shapes, attributed to biaxial growth during the expansion of the foam [36]. It could be observed that the samples with linear and triangular patterns at 20% infill presented smoother surfaces, while those with 80% infill presented bubbles on the surface of the material. It should be mentioned that for scalable foam production, these materials can be introduced in a grid mold to be further foamed in the desired shape.

On the other hand, while the weight of the foamed samples did not change significantly during the foaming process, the thickness of the sample was modified as expected. In this regard, it can be observed in Table 3 that the foams obtained presented an average thickness 2.0 to 2.5 times greater than the 3D-printed parts. This result was aligned with prior research conducted on PLA parts and PLA bionanocomposites foamed by sc-CO_2_ [31,36].

### 3.2. Viscosity Molecular Weight

Table 4 shows a summary of the estimated viscosity molecular weight of both the PLA filament and 3D-printed parts and foams. The estimated viscosity molecular weight (M_v_) of the commercial PLA filament falls within a similar range as that previously determined PLA pellet, approximately ~186,000 g/mol [31].

The materials developed herein were prepared with the PLA filament subjected to two different infill printing processes of 20% and 80% as well as using two infill types, linear and triangular. The 3D-printed materials exhibited a significant decrease in viscosity molecular weight, attributed to chain scission resulting from thermal degradation during the thermal 3D-printing process. The decrease in the viscosity and in the molecular weight due to PLA deterioration during the FDM process has been previously documented in 3D-printed samples by Zhao et al. [42]. In the present research, it was observed that the 80% infill produced a higher reduction of the viscosity molecular weight of the PLA filament (between 23% and 26% reduction) than the 20% infill (between 13% and 16% of reduction) probably due to the higher time that the PLA filament is subjected to a 3D-printing process in the case of 80% of infill since the material is exposed to a high-temperature process (220 °C) and experienced higher thermal degradation.

In the case of the foamed samples, it was noted that the scCO_2_ process leads to a reduction in the molecular weight of the 3D-printed samples, akin to the effect observed during the printing process but less marked (*p* < 0.05). Although this reduction was somewhat more pronounced in the case of the sample with 80% infill (*p* > 0.05), ranging between 8.5% and 9.5%, the main reduction in the viscosity molecular weight occurred during the 3D-printing process. The low reduction observed in the viscosity molecular weight of PLA-based foams processed by scCO_2_ has been already observed in our previous works [31,36]. The obtained results highlight the interest in using the scCO_2_ process to easily produce foamed materials from 3D-printed parts.

### 3.3. Morphological Analysis

**Figure 4:**
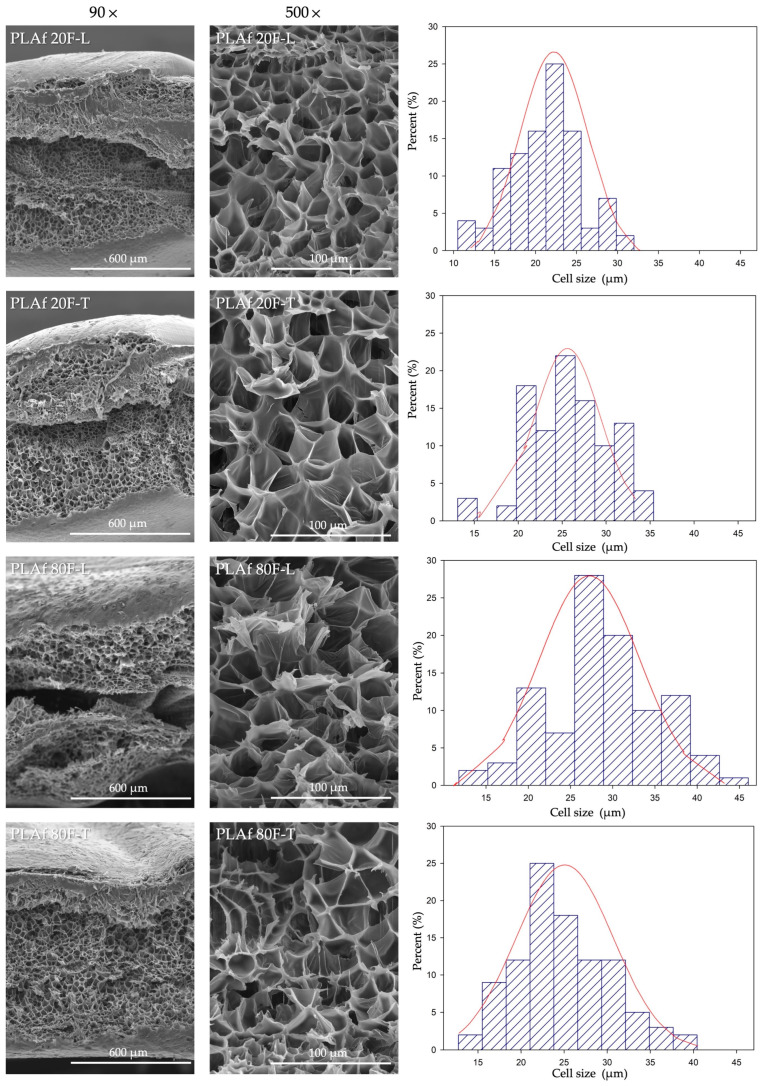
Scanning electron microscopy (SEM) images of the surface, cross-sections, and cell size porous distribution of 3D-printed PLA foams.

Figure 4 shows SEM micrographs depicting the cross-section of the freeze-fractured surface of all PLA foams obtained through 3D printing. The figure exhibits that all samples presented a traditional closed cell structure, with higher expansion ratios, higher for those samples with 80% infill (PLAf 80RP-L and PLAf 80RP-T) and a much more uniform cell distribution. In detail, the samples with 80% infill presented triangular-shaped cells, due to which the void fraction is increased [43]. This is concordant with the data obtained for the viscosity molecular weight, i.e., the samples with lower viscosity molecular weight presented a larger mean cell size, meaning the PLAf 80F-L foam with a mean cell value of 28.79 μm, as shown in Figure 4. For the PLAf 20F-L foam, characterized by a greater molecular weight, the corresponding mean cell size was 21.08 μm, as depicted in the figure.

Although the influence of CaCO_3_ as PLA reinforcement on the crystallization and rheological properties of PLA-based foams is widely reported [44,45,46,47], the effect of CaCO_3_ on foamability during scCO_2_ foaming of 3D-printed parts has not been described. In this work, since no agglomerations were observed, it can be concluded that the presence of CaCO_3_ did not influence the surface of the foams or the cell walls. Nevertheless, it is essential to note that these particles may serve as nucleation sites for cells during the foaming process, hindering gas absorption but aiding in cell nucleation. Furthermore, the material’s microcellular morphology is significantly influenced by the composite composition (PLA and CaCO_3_) and the foaming processing conditions (pressure and temperature) [48].

### 3.4. FTIR Analysis

**Figure 5:**
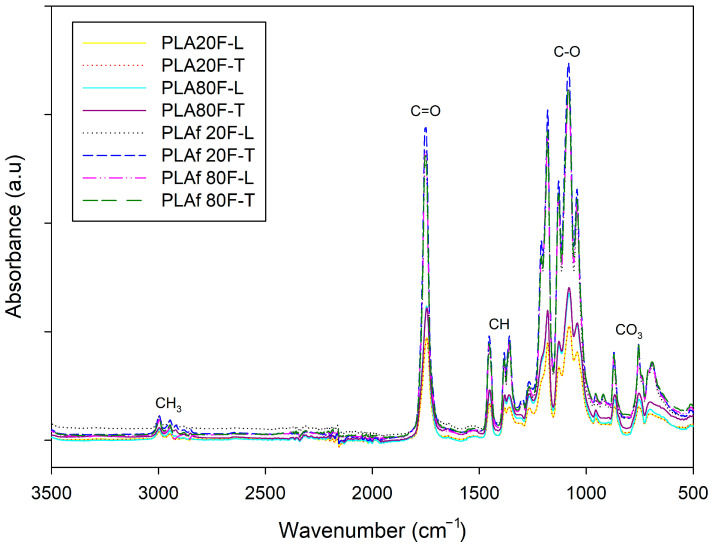
FTIR spectra of 3D-printed parts and PLA foams.

Figure 5 presents the FTIR spectra of 3D-printed parts and PLA foams which were used to recognize the different functional groups present in each material. The characteristic peaks of PLA at 2944 cm^−1^ and 2995 cm^−1^ can be appreciated and are ascribed to symmetrical and asymmetrical vibrations of axial -CH groups in saturated hydrocarbons (CH_3_) [31]. The peaks observed at 1746 cm^−1^ and 1084 cm^−1^ are respectively attributed to the stretching of C=O in the carbonyl ester and the bending vibrations of the ether bond [49]. While the peaks at 920, 1380, and 1359 cm^−1^ are ascribed to symmetrical and asymmetrical vibrations of CH bonds in the CH_3_ of PLA [50], the peaks of the C-O stretching bond are centered at 1042 cm^−1^, 1082, and 1128 cm^−1^ [51], and the peaks at 957 cm^−1^, and 870 cm^—1^ have been ascribed to the amorphous and crystalline PLA phases, respectively [8], attributed to the characteristic vibrations of the helical backbone with CH_3_ rocking modes [52]. The band related to the PLA amorphous regions (957 cm^−1^) appeared in both PLA-based films and foams. Meanwhile, the band centered at 920 cm^−1^, related to the PLA crystalline regions only appeared in PLA-based foams, showing that the sc-CO_2_ foaming process is able to crystallize the PLA polymeric matrix. Then again, the most characteristic peaks of calcium carbonate-CaCO_3_ are also observed, such as the case of the peaks centered at 1795 and 1450 cm^−1^, which are attributed to C-O stretching vibration, as well as the peaks at 713 and 875 cm^−1^, related to the strong C-O bending vibration of CO_3_^2−^ as presented [53,54].

**Figure 6:**
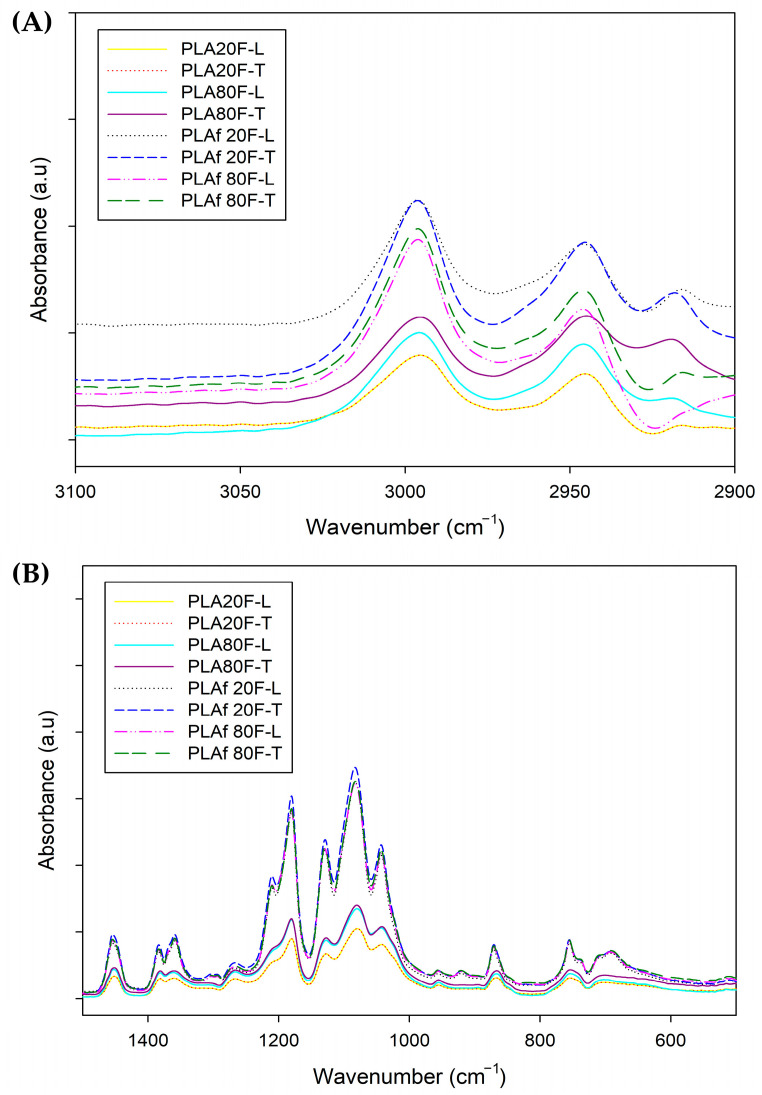
FTIR spectra between (**A**) 2900 cm^−1^ and 3100 cm^−1^ as well as (**B**) 600 cm^−1^ and 1500 cm^−1^ of 3D-printed parts and PLA foams.

Figure 6 shows that PLA parts printed with an infill of 20% and 80% of rectilinear and triangular patterns did not present modifications in the chemical structure. However, it can be seen there are variations in intensity (Figure 6A), the parts obtained present a directly proportional relationship between the intensity and the percentage of infill, in the samples with an infill of 80% the intensity increases compared to the parts with infill of 20%. In the foamed samples, there is a notable increase in crystallinity, as indicated in Figure 6B. The FITR spectrum showed absorption peaks around 1200 cm^−1^, associated with the vibration of the alkyl ketone chain, and at 920 cm^−1^, linked to the bending vibration of the C-H bond, representing the crystalline structure [55]. The foaming process of polymer is closely related to crystallization. The crystals enhance the local supersaturation of CO_2_, they provide heterogeneous nucleation sites for bubble nucleation, and the existence of crystals can effectively inhibit the coalescence and rupture of bubbles during the bubble growth stage [56,57]. Analyzing the FTIR, between the different samples, the foams exhibit an intensified signal across all samples (Figure 6A). This intensification can be attributed to the pressure applied during the measurement, highlighting a direct correlation between pressure and intensity. In simpler terms, as the volume expands, the applied pressure increases, leading to a subsequent rise in peak intensity. The expansion of internal space within the samples results in a heightened absorption of the emitted light beam, consequently amplifying the intensity of the characteristic peaks observed in the foams.

### 3.5. Thermal Properties

**Figure 7:**
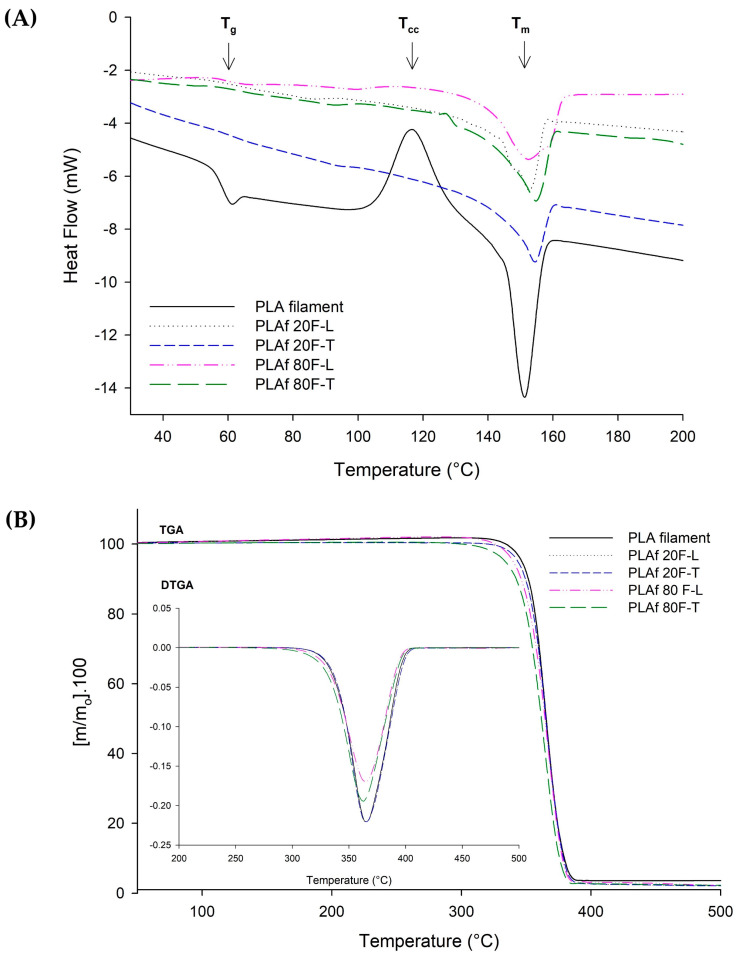
Thermal results: (**A**) DSC and (**B**) TGA and DTGA (their derivative) curves of 3D-printed PLA foams.

Figure 7A shows the DSC thermograms acquired during the first heating process. In the PLA filament sample, the glass transition temperature was observed at around 62 °C, followed by a cold crystallization temperature at about 114 °C, and a melting peak at approximately 154 °C [10]. Similar findings were observed for the 3D-printed parts as can be seen in PLA20F-T as an example (see Figure S1).

Concerning PLA foams printed with 80% and 20% infill, using both rectilinear and triangular patterns, there was no evidence of cold crystallization temperature. In all samples, only the melting peak, occurring around 150 °C, was observed. This implies that the material underwent complete crystallization during the foaming process with supercritical CO_2_ [36], in good agreement with the FTIR results.

Figure 7B illustrates the TGA curves and corresponding TGA parameters presented in Table 5 for both the PLA filament and the newly developed PLA foams. The results indicate thermal stability in the samples up to approximately 300 °C, with a consistent maximum degradation temperature (T_max_) of around 365 °C for all samples. Notably, the TGA parameters reveal that the degradation of all samples initiates in a single step around 330 °C. The initiation temperature for 5% (Td, 5%) was at approximately 335 °C, and in all instances, complete decomposition occurred above 400 °C. 

These results align well with those previously reported [21,36,58,59]. However, in the foamed samples with an infill of 80%, PLAf 80F-L and PLAf 80F-T, subtle temperature fluctuations are observed, as detailed in Table 5. The reduction in temperature present in these materials could be attributed to the 3D-printing technique, specifically in the infill percentage, which corresponds to the amount of plastic versus the total volume of the 3D model. That is, as the infill percentage increases, the available space inside the printed piece decreases. Pieces with an infill percentage above 90% exhibit behavior very similar to injection-molded pieces [60,61]. Consequently, when using an infill of 80%, solid and uniform material samples are produced. Certainly, a slight decrease in the thermal stability of the material was observed with 80% infill. This situation could potentially impact the material properties, such as thermal conductivity or thermal resistance. In the case of homogeneous materials thermal resistance is the ratio of the thickness to the thermal conductivity of the material, that is, the ability of materials to oppose the passage of heat. It is important to mention that homogeneous material could require a lower temperature to degrade because the uniform distribution of the infill would affect the thermal response of the material.

### 3.6. Tensile Properties

**Figure 8:**
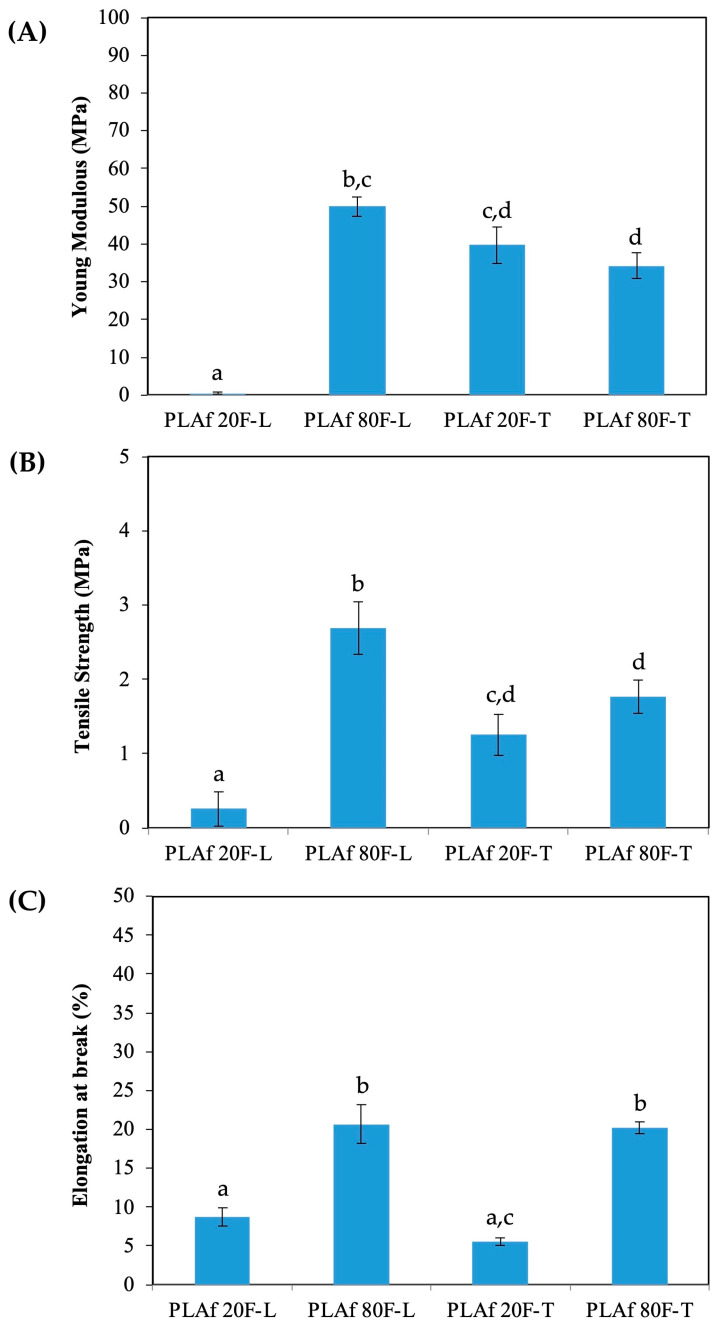
Tensile properties of 3D-printed PLA foams from tensile test measurements. (**A**) Young Modulus, (**B**) Tensile Strength and (**C**) Elongation at break. ^a–d^ Different superscripts within the same bar indicate significant differences (*p* < 0.05).

Figure 8 shows the mean results derived from tensile test analysis. It is evident that, although two percentages of infill within the triangular pattern exhibit similar values, there is a substantial disparity in the results for the rectilinear inner pattern based on the infill amount. Specifically, both the PLAf 20R-T and PLAf 80R-T samples demonstrated Young’s modulus and tensile strength values of around 35 MPa and 2 MPa, respectively.

For the elongation at break, results were significantly different being around 5% for PLAf 20R-T and around 80% for PLAf 80R-T. However, for the rectilinear pattern samples, the average values obtained for 20RPL are extremely low, which is significantly enhanced by increasing the infill to 80% (PLAf 80R-L). This suggests that in the mechanical performance of foams obtained from 3D-printed parts followed by scCO_2_, the infill percentage is a very significant parameter that depends on the type of printing pattern that was selected.

Table 6 presents the numerical values obtained from tensile tests for both the 3D-printed parts and the corresponding foamed 3D-printed parts. In general terms, the values observed in the 3D-printed parts depend directly on the percentage of infill regardless of the printing pattern selected, as the adhesion between layers is lower with less infill which may lead to flaws and mechanical limitations [4]. The greater the amount of infill, the more Young’s modulus increases from values close to 400 MPa to 1300–1700 MPa depending on the pattern, and the strength decreases, although slightly. 

On the other hand, the increase in infill percentage does not significantly impact the elongation at break. However, the linear pattern type showed higher values compared with the parts with the triangular pattern. This is probably due to the linear pattern, which consists of a parallel pattern, and hence produces inner filaments easily oriented to the tensile effort.

Regarding the values of the 3D-foamed samples, tensile test results are notably lower than those obtained for its 3D-printed part counterparts. When the 3D-printed parts are subjected to the foaming process, the diffusion of supercritical CO_2_ particles together with the conditions of temperature and pressure, the internal composition of the material becomes porous in its structure. The loss of structural continuity leads to a noticeable decrease in modulus and strength, which both reach extremely low values while the elongation at break increases significantly. As expected, for lower infill contents, the foams obtained show lower tensile behavior. However, at least in this work, for lower infill content, 3D pieces with a triangular pattern are less affected in terms of tensile properties when they are foamed, compared to the linear pattern. This may be due to the greater number of nodes that are formed internally in pieces printed with the triangular pattern. When an area is filled with a series of intersecting triangles, obviously the number of nodes is greater than if the same area is filled with parallel lines. Therefore, when the biaxial growth caused by foaming with supercritical CO_2_ occurs, a greater number of points where several segments join allow a more compact structure where inner stresses can be spread more adequately. These results are in agreement with those obtained previously in the foaming of foamed PLA bionanocomposites, where the results reported were values close to those obtained in this work [36].

## 4. Conclusions

Successfully, 3D-printed PLA/CaCO_3_ foams were obtained through a simple foaming method utilizing supercritical CO_2_, starting from 3D-printed parts produced by FDM. It was observed that all the 3D-printed materials showed a significant reduction in the viscosity molecular weight ascribed to the chain scission produced by thermal degradation during the thermal 3D-printing process. Meanwhile, the scCO_2_ process induces a minimal decrease in the viscosity molecular weight of the 3D-printed samples, notably in those with 80% infill (reduction between 8.5% and 9.5%). This underscores the potential of utilizing scCO_2_ for foam production.

SEM analysis demonstrated that closed-cell foams were acquired. FTIR results showed that all the samples present characteristic peaks of PLA polymeric matrix and calcium carbonate-CaCO_3_ along the spectrum, both in the 3D-printed parts and in the foams. Foams show significant crystallinity recovery, linked to polymer foaming and crystallization processes, evidenced by characteristic FTIR signals correlated with applied pressure and volume expansion. These findings were corroborated by DSC thermal results, which showed that PLA foams printed with an infill of 20% and 80% of rectilinear and triangular patterns did not present the cold crystallization temperature, showing that the material crystallized completely during the foaming process with supercritical CO_2_. TGA curves showed that samples were thermally stable up to ∼300 °C. Subtle temperature fluctuations in 80% infill foamed samples were shown. The 80% infill reduces thermal stability, potentially impacting material properties. Homogeneous materials with such infill may require lower temperatures for degradation due to altered thermal response. Nevertheless, the materials were thermally stable at the temperature for the intended use.

Tensile results suggested that the infill percentage is a very significant parameter for these foams as well as the type of printing pattern. For lower infill percentages, the foams obtained show lower tensile behavior. However, for lower infill content pieces with a triangular pattern are less affected in terms of tensile properties when they are foamed, compared to the linear pattern. But for foamed 3D-printed parts with an infill percentage of 80%, a greater number of nodes are formed internally in pieces printed with the triangular pattern than in the case of the linear pattern.

The results acquired here highlight the interest in using the scCO_2_ process to easily produce foams from 3D-printed parts. According to the obtained results, it could be concluded that the foamed 3D-printed parts using 80% infill and designed by a triangular infill pattern may offer a sustainable alternative to replace non-biodegradable materials such as Expanded Polystyrene (EPS) and they are a promising option for use in many industrial sectors and in numerous applications, such as food contact materials and other single-use goods.

## Figures and Tables

**Table 1:** Assignment of 3D-printed parts in this work, type, and the infill percentage employed.

Sample	Infill Percentage	Type of Infill
20F-L	20%	Rectilinear-L
20F-T	20%	Triangular-T
80F-L	80%	Rectilinear-L
80F-T	80%	Triangular-T

**Table 2:** Operational parameters for the 3D FDM printing process.

Parameters	Values
Speed of nozzle movement	1800 mm/min; first layer 300 mm/min
Nozzle temperature	220 °C
Bed temperature	Room temperature
Layer height	0.1–0.3 mm
Extrusion width	0.48 mm

**Table 3:** Mean values for thickness of parts and foams.

Sample	Thickness (mm)
3D-parts	
PLA 20F-L	0.605 ± 0.045
PLA 20F-T	0.644 ± 0.022
PLA 80F-L	0.637 ± 0.042
PLA 80F-T	0.667 ± 0.036
Foams	
PLAf 20F-L	1.359 ± 0.082
PLAf 20F-T	1.824 ± 0.190
PLAf 80F-L	1.494 ± 0.151
PLAf 80F-T	1.830 ± 0.155

**Table 4:** Estimated Viscosity Molecular Weight (Mv).

Sample	Molecular Weight (g mol^—1^)
PLA filament	192,200 ± 6700 ^a^
3D-parts	
PLA20F-L	160,900 ± 4000 ^b^
PLA20F-T	166,500 ± 2000 ^b^
PLA80F-L	146,700 ± 1100 ^b,c^
PLA80F-T	142,100 ± 2900 ^c^
Foams	
PLAf 20F-L	153,000 ± 8200 ^b,c^
PLAf 20F-T	136,300 ± 10,000 ^b,c^
PLAf 80F-L	134,100 ± 600 ^c^
PLAf 80F-T	128,600 ± 4100 ^c^

^a–c^ Different superscripts indicate significant differences (*p* < 0.05).

**Table 5:** TGA parameters of PLA filament and 3D-printed PLA foams.

Sample	T_onset_ (°C)	T_d (5%)_ (°C)	T_max_ (°C)	T_endset_ (°C)
PLA filament	333.3	346.3	364.5	393.4
PLAf 20F-L	335.6	339.7	365.1	396.5
PLAf 20F-T	335.5	339.7	365.5	393.6
PLAf 80F-L	327.8	337.6	360.0	388.9
PLAf 80F-T	331.1	325.7	362.3	392.8

**Table 6:** Tensile test results of the 3D-printed parts and the 3D-printed foams.

Sample	E (MPa)	TS (MPa)	$\varepsilon_{b}$
3D-parts			
PLA 20F-L	450.6 ± 25.1 ^a^	22.5 ± 2.7 ^a^	8.2 ± 1.4 ^a^
PLA 80F-L	1,308.3 ± 51.4 ^b^	18.9 ± 2.7 ^a^	8.3 ± 1.8 ^a^
PLA 20F-T	326.4 ± 58.7 ^c^	12.1 ± 1.7 ^b^	3.9 ± 0.3 ^a,b^
PLA 80F-T	1,719.5 ± 51.3 ^d^	6.6 ± 0.5 ^c^	1.9 ± 0.4 ^b^
Foams			
PLAf 20F-L	0.5 ± 0.2 ^e^	0.5 ± 0.6 ^d^	11.3 ± 1.2 ^a^
PLAf 80F-L	43.1 ± 8.6 ^f^	2.3 ± 0.5 ^e^	28.9 ± 2.4 ^c^
PLAf 20F-T	31.9 ± 6.5 ^f^	1.3 ± 0.2 ^f^	7.7 ± 0.4 ^a^
PLAf 80F-T	34.3 ± 3.5 ^f^	1.8 ± 0.2 ^f^	26.4 ± 3.6 ^c^

^a–f^ Different superscripts within the same column indicate significant differences (*p* < 0.05).

## Data Availability

The data presented in this study are available on request from the corresponding authors.

## Supplementary Materials

The following supporting information can be downloaded at: https://www.mdpi.com/article/10.3390/polym16060798/s1, Figure S1: DSC curves of 3D-printed PLA20F-T sample.
