# Supplementary material for: Foaming of 3D-Printed PLA/CaCO3 Composites by Supercritical CO2 Process for Sustainable Food Contact Materials
Source: Polymers (Basel). 2024 Mar 13;16(6):798. doi: 10.3390/polym16060798 (PMC10974494; doi:10.3390/polym16060798)
Supplement: Supplementary file 1 [file polymers-16-00798-s001.zip › polymers-2825066-supplementary.pdf]

polymers

MDPI

Supporting Information

Foaming of 3D printed PLA/$CaCO_3$ composites by Supercritical CO_2 process for sustainable food contact materials

Simón Faba [1,2*], Ángel Agüero [2,3], Marina P. Arrieta [2,4,*], Sara Martínez [1], Julio Romero [5], Alejandra Torres [1] and María José Galotto [1]

1 Packaging Innovation Center (LABEN-CHILE), Department of Food Science and Technology, Faculty of Technology, Center for the Development of Nanoscience and Nanotechnology (CEDENNA), University of Santiago de Chile (USACH) 9170201 Santiago, Chile.; simon.faba@usach.cl (S.F.); maria.galotto@usach.cl (M.J.G)
2 Departamento de Ingeniería Química Industrial y del Medio Ambiente, Escuela Técnica Superior de Ingenieros Industriales, Universidad Politécnica de Madrid (ETSII-UPM), Calle José Gutiérrez Abascal 2, 28006 Madrid, Spain.; m.arrieta@upm.es (M.P.A.)
3 Instituto Universitario de Tecnología de Materiales (IUTM), Universidad Politécnica de Valencia (UPV), Plaza Ferrándiz y Carbonell 1, Alcoy 03801, Spain; anagrod@epsa.upv.es (Á.A.)
4 Grupo de Investigación: Polímeros, Caracterización y Aplicaciones (POLCA), 28006 Madrid, Spain.; m.arrieta@upm.es (M.P.A.)
5 Laboratory of Membrane Separation Processes (LabProSeM), Department of Chemical Engineering and Bioprocesses, Engineering Faculty, University of Santiago de Chile (USACH), Santiago 9170201, Chile; julio.romero@usach.cl (J.R.)

* Correspondence: m.arrieta@upm.es (M.A.); simon.faba@usach.cl (S.F.)

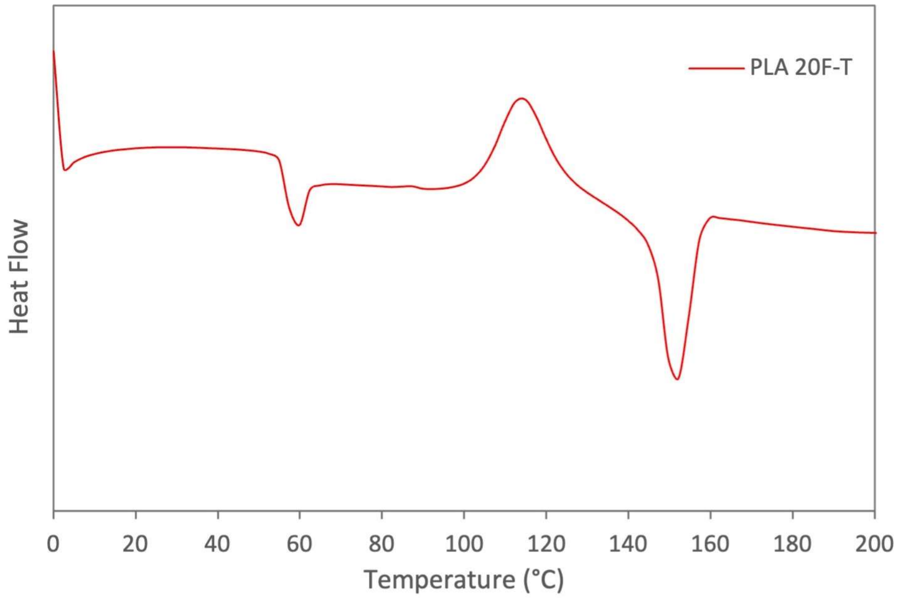

Figure S1. DSC curves of 3D printed PLA20F-T sample.
